# Supplementary material for: Cognitive subgroups of affective and non-affective psychosis show differences in medication and cortico-subcortical brain networks
Source: Sci Rep. 2024 Sep 2;14:20314. doi: 10.1038/s41598-024-71316-3 (PMC11369100; doi:10.1038/s41598-024-71316-3)
Supplement: Supplementary file 1 — Supplementary Information. [file 41598_2024_71316_MOESM1_ESM.pdf]

Supplementary Material: Cognitive subgroups of affective and non-affective psychosis show differences in medication and cortico-subcortical brain networks

Katharina M Bracher[1], Afra Wohlschlaeger[2], Kathrin Koch[2], Franziska Knolle[2],*

[1]) Division of Neurobiology, Faculty of Biology, LMU Munich, Martinsried 82152, Germany

[2]) Department of Diagnostic and Interventional Neuroradiology, School of Medicine, Technical University of Munich, Munich, Germany

* Corresponding Author: franziska.knolle@tum.de

Conflict of Interest: None of the authors declares a conflict of interest.

Author contributions: FK, KB, AW conceptualisation; KK preprocessing of structural brain data; FK and KB formal analysis of cognitive data and clustering analysis; FK and KB writing of first draft; FK, KB, AW and KK editing and revising of manuscript.

Acknowledgement: Research using Human Connectome Project for Early Psychosis (HCP-EP) data reported in this publication was supported by the National Institute of Mental Health of the National Institutes of Health under Award Number U01MH109977. The HCP-EP 1.1 Release data used in this report came from DOI: 10.15154/1522899.

Data availability statement: All processed data and code may be accessed via: `https://github.com/KatharinaBracher/earlypsychosis_clustering`

A Data

A.1 Patients

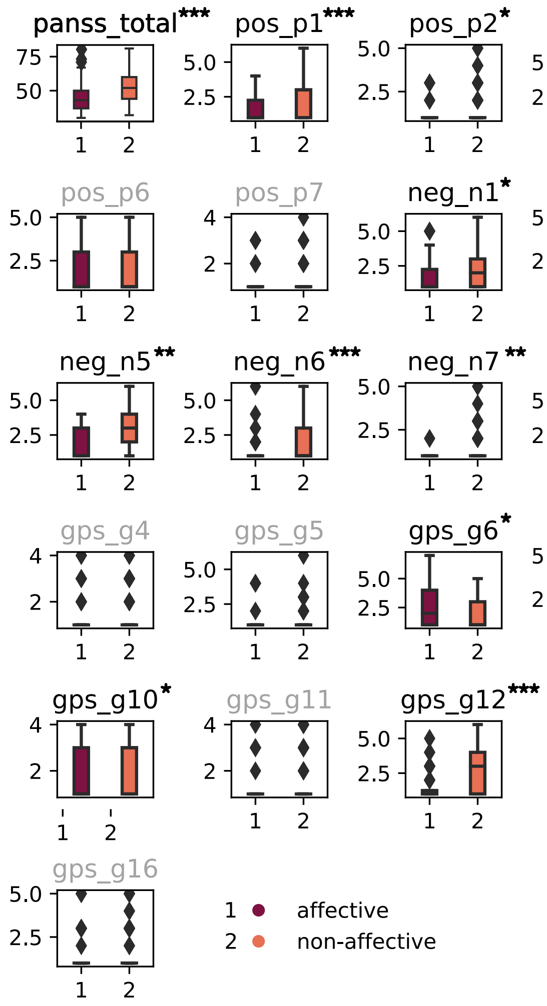

Suppl. Figure 1: **Comparison of patient PANSS items.** Individual boxplots represent the distribution of each PANSS item for affective (1) and non-affective (2) patients, showing data minimum, first quartile, median, third quartile, and data maximum. Outliers are indicated outside the minimum or maximum. Significant differences between affective and non-affective are indicated with stars, where * corresponds to $p < 0.05$, ** to $p < 0.01$ and *** to $p < 0.001$ (using a Kruskal-Wallis H-test).

A.2 Cognitive Data

Suppl. Table 1: Features and corresponding description that were considered for our analysis. Of 70 cognitive features, 37 were excluded due to missing entries, which is displayed in the last column where + corresponds to included in analysis and - to excluded of analysis.

Feature	Description	included
acpt01_%Hits	Total Q3A-Block : %Hits	+
acpt01_RT	Total Q3A-Block : RT	+
cgi01_gaf2a	Participant and clinician report-Symptom Scale Score	+
cgi01_gaf2b1	Participant and clinician report-Occupational Functioning Scale Score	+
cgi01_gaf2b2	Participant and clinician report-Occupation	+
cgi01_gaf2c	Participant and clinician report-Social Functioning Scale Score	+
cogcomp01_nih_fluidcogcomp_unadjusted	Fluid Cognition Composite Score unadjusted	+
cogcomp01_nih_crycogcomp_unadjusted	Crystal Cognition Composite Score unadjusted	+
dccs01_acc	Accuracy	-
dccs01_rt	Reaction Time	-
dccs01_nih_dccs_ageadjusted	Age Adjusted scaled score for DCCS subtest	+
deldisk01_auc_200	Area Under the Curve for Delay Amount $200	+
deldisk01_auc_40000	Area Under the Curve for Delay Amount $40000	+
er4001_er40_c_cr	Correct Responses	-
er4001_er40_c_rtcr	Correct Responses Median Response Time (ms)	-
er4001_er40_c_ang	Correct Anger Identifications	-
er4001_er40_c_fear	Correct Fear Identifications	-
er4001_er40_c_hap	Correct Happy Identifications	-
er4001_er40_c_noe	Correct Neutral Identifications	-
er4001_er40_c_sad	Correct Sad Identifications	-
flanker01_acc	Accuracy	-
flanker01_rt	Reaction Time	-
flanker01_nih_flanker_ageadjusted	Age Adjusted scaled score for Flanker subtest	+
lswmt01_nih_tlbx_tscore	T-score	-
lswmt01_nih_tlbx_agegencsc	Age-Gender-Corrected T-score	-
lswmt01_nih_tlbx_fctsc	Fully-Corrected T-Score	-
lswmt01_tbx_ls	List Sort Test total score	+
orrt01_read_acss	Reading test Age-Corrected Standard Score	+
orrt01_read_fcts	Reading test Fully Corrected T-score	-
pcps01_nih_tlbx_fctsc	Fully-Corrected T-Score	-
pcps01_nih_patterncomp_ageadjusted	Age Adjusted scaled score for PatternComp subtest	+
prang01_anger_ts_nih_toolbox_anger-physical_aggression_ff_age_18+_v2.0	Anger T score	+
prang01_anger_ts_nih_toolbox_anger-hostility_ff_age_18+_v2.0	Anger T score	+
prang01_anger_ts_nih_toolbox_anger-affect_cat_age_18+_v2.0	Anger T score	+
predd01_edd_rs	Emotional Distress Depression raw score	+
prsi01_soil_rs	Social Isolation raw score	+
psm01_nih_picseq_ageadjusted	Age Adjusted scaled score for PicSeq subtest	+
pss01_pss_distress_rs	PSS Distress raw score	+
self_effic01_nih_tlbx_rawscore	RawScore	+
tlbx_emsup01_nih_tlbx_rawscore_nih_toolbox_instrumental_support_ff_age_18+_v2.0	RawScore	+
tlbx_emsup01_nih_tlbx_rawscore_nih_toolbox_emotional_support_ff_age_18+_v2.0	RawScore	+

Feature	Description	included
tlbx_friend01_nih_tlbx_rawscore	RawScore	+
tlbx_perhost01_nih_tlbx_rawscore	RawScore	+
tlbx_rej01_pr_score	Peer Rejection Score	+
tlbx_wellbeing01_tlbxpa_ts	Positive affect t-score	+
tpvt01_tlbx_readncorr	English Reading Number Correct	-
tpvt01_lavoc_screen	Vocabulary Starting theta based on age/education	-
tpvt01_tpvt_acss	TPVT age-corrected standard score	+
tpvt01_tpvt_fcts	TPVT fully corrected T-score	-
wasi201_ss_blockdesigntscoreperf4	Block Design T - Score Performance - 4 Subtest	-
wasi201_ss_vocabularytscoreverbal4	Vocabulary T - Score Verbal - 4 Subtest	-
wasi201_ss_vocabularytscore2	Vocabulary T - Score - 2 Subtest	-
wasi201_ss_matrixreasoningtscoreperf4	Matrix Reasoning T - Score Performance - 4 Subtest	-
wasi201_ss_matrixreasoningtscore2	Matrix Reasoning T - Score - 2 Subtest	-
wasi201_ss_similaritiestscoreverbal4	Similarities T - Score Verbal - 4 Subtest	-
wasi201_sumstscores_verbal4subtest	Verbal - Score - 4 Subtest	-
wasi201_sumstscores_perf4subtest	Performance - Score - 4 Subtest	-
wasi201_sumstscores_total4subtest	Total - Score - 4 Subtest	-
wasi201_sumstscores_total2subtest	Total - Score - 2 Subtest	-
wasi201_iqscores_verbsumtscores	Verbal Sum of T - Scores	-
wasi201_iqscores_verbiq	Verbal IQ	-
wasi201_iqscores_verbpercentile	Verbal Percentile	-
wasi201_iqscores_verbconfintervalfrom	Verbal Confidence Interval - From	-
wasi201_iqscores_verbconfintervalto	Verbal Confidence Interval - To	-
wasi201_iqscores_perfsumtscores	Performance Sum of T - Scores	-
wasi201_iqscores_perfiq	Performance IQ	-
wasi201_vocab_totalrawscore	Total Raw Score - Vocabulary	+
wasi201_matrix_totalrawscore	Total Raw Score - Matrix Reasoning	+
wasi201_iqscores_full2iq	Full 2 IQ	+
ymrs01_ymrstot	YMRS Total score	-

Suppl. Table 2: Comparison between patients and control subjects for cognitive features that contributed most to variance, as well as medication and PANSS comparison between diagnosis. For the group comparison, a Kruskal-Wallis was used with a Dunn's test for pairwise comparisons (statistics and p-value displayed or ns - non significant).

Feature	group comparison	contr. - affect.	contr. - non-aff.	affect. - non-aff.
**Cognitive scores**				
Fluid cognition	46.1196, <0.001	8.6884, <0.01	42.3859, <0.001	12.5001, <0.001
Crystallized cognition	23.732, <0.001	ns	20.3015, <0.001	8.7481, <0.01
Total IQ	34.7911, <0.001	ns	29.809, <0.001	13.3756, <0.001
Picture vocabulary test	23.1115, <0.001	ns	18.5334, <0.001	10.6137, <0.01
Oral reading recognition	17.7379, <0.001	ns	16.3271, <0.001	4.3483, <0.05
Auditory attention %correct	43.5029, <0.001	10.1902, <0.01	40.613, <0.001	9.9483, <0.01
Working memory	30.1556, <0.001	ns	22.7316, <0.001	16.1795, <0.001
WASI - Verbal comprehension	19.9352, <0.001	ns	16.0612, <0.001	9.6666, <0.01
WASI - Matrix reasoning	34.7254, <0.001	ns	30.1583, <0.001	12.0238, <0.001
DCCS - Executive functioning	23.4622, <0.001	4.9034, <0.05	21.395, <0.001	6.2001, <0.05
**Medication status**				
Lifetime Antipsychotic Drug Exposure (months)	-	-	-	24.4946, <0.001
Chlorpromazine Equivalence (mg/d)	-	-	-	18.7923, <0.001
Scan Date Chlorpromazine Equivalence (mg)	-	-	-	17.6053, 0.0
**Positiv negative symptom score**				
PANSS total	-	-	-	14.71, <0.001
PANSS positiv	-	-	-	9.09, <0.01
PANSS negativ	-	-	-	9.44, <0.01
general psychopathology	-	-	-	7.01, <0.01
**Brain clusters**				
NW 18	7.708, <0.05	ns	7.4014, <0.01	ns
NW 29	-	-	-	-
NW 30	7.9038, <0.05	ns	7.7348, <0.01	ns

Suppl. Table 3: Features with number of missing individuals. Missing values for continuous features were replaced by the mean according to the group of patients or controls. For categorical data, the mode was used to replace the missing value. *W* stands for 'white', for Socio-Economic Status, 2 for a SES score of 20 to 29 and in the Mother/Father Educational Scale a 4 for High School Graduation or GED and a 6 for Completed 7th through the 9th grades.

Feature	Missing con. + pat.	Controls mean (std)	Patients mean (std)
**Demographics**			
Socio-Economic Status	0+2	2	2
Mother Educational Scale	1+6	6	6
**Auditory continuous performance test**			
%Hits	1+3	91.0 (6.89)	81.29 (13.29)
Reaction time	1+3	623.19 (72.38)	669.07 (104.61)
**Cognition Composite Scores**			
Fluid Cognition Composite Score unadjusted	0+2	112.34 (11.22)	100.07 (13.09)
Pattern Comparison Processing Speed - Age adjusted	0+1	104.32 (20.44)	91.54 (21.08)
**Delay Discounting Task**			
Area Under the Curve Delay Amount $200	0+1	0.27 (0.26)	0.25 (0.26)
Area Under the Curve for Delay Amount $40000	0+1	0.55 (0.28)	0.43 (0.32)
**PROMIS Anger**			
Physical Aggression Anger T score	0+3	49.25 (9.15)	55.65 (11.17)
Hostility Anger T score	0+3	52.14 (9.45)	57.68 (10.21)
Affect Anger T score	0+3	45.8 (10.62)	50.94 (14.2)
**NIH Toolbox Sadness CAT Age 18+ v2.0**			
Emotional Distress Depression raw score	0+3	9.34 (2.55)	11.86 (6.74)
**NIH Toolbox Sadness CAT Age 18+ v2.0**			
Age Adjusted scaled score for PicSeq subtest	0+1	106.71 (14.16)	92.91 (15.27)
**NIH Toolbox Sadness CAT Age 18+ v2.0**			
Instrumental Support Raw Score	0+3	32.29 (6.27)	29.46 (8.36)
Emotional Support Raw Score	0+3	34.25 (7.12)	30.98 (7.07)
**NIH Toolbox Emotion Domain - Psychological Well-Being**			
Positive affect t-score	0+3	49.45 (6.9)	43.55 (8.82)

Suppl. Table 4: Number of missing features per subject

Subject ID	Number of missing features
1057	1
1063	4
1068	2
1099	1
1118	2
2005	7
2007	7
2012	2
2023	7
3031	2
4053	1
4059	1
4063	1
4065	2
4092	2
1015	2

A.3 Brain Data

NW1:

1: 6932, 14, -66, -48, cerebellum VIIIa

2: 6590, -20, -69, -60, cerebellum VIIIa, cerebellum crus

NW2:

1: 6763, 8, -48, -28, cerebellum VI

2: 5574, -33, -51, -39, cerebellum VI

NW3:

1: 7340, -18, -70, -28, cerebellum VI, cerebellum crus I

NW4:

1: 11747, 2, -57, -27, cerebellum I-V

2: 1168, -16, -40, -58, cerebellum VIIIb

3: 134, 21, -42, -56, cerebellum VIIIb

NW5:

1: 10338, 6, -74, -38, cerebellum VIIb, cerebellum crus

NW6:

1: 5836, -18, -2, -33, parahippocampal gyrus, temporal pole

2: 3948, 34, -6, -51, parahippocampal gyrus, temporal pole

3: 329, -21, -87, -46, cerebellum crus II

NW7:

1: 10263, -2, -56, -46, r. cerebellum IX, l. cerebellum IX

2: 489, -27, -56, -36, cerebellum VI, cerebellum V

NW8:

1: 6947, -42, -60, -56, cerebellum VIIb, cerebellum crus

2: 5665, 40, -58, -51, cerebellum VIIb, cerebellum crus

3: 352, -14, -6, 10, thalamus

NW9:

1: 6039, -40, -4, -38, fusiform, middle temporal gyrus, inferior temporal gyrus

2: 5371, 48, -10, -46, middle temporal gyrus, inferior temporal gyrus

NW10:

1: 6981, 3, -78, 8, intracalcarine cortex, lingual gyrus

2: 177, 32, -82, 26 lateral occipital cortex

NW11:

1: 12704, 0, 20, 27, cingulate gyrus

2: 176, -33, 33, 33, middle frontal gyrus

3: 109, 34, 27, 30, middle frontal gyrus

4: 100, -8, -4, 15, thalamus

NW12:

1: 7408, 2, -64, 18, precuneus, supracalcarine cortex

NW13:

1: 5803, -8, -66, 22, precuneus

2: 1015, -28, -62, -33, cerebellum VI, cerebellum crus I

3: 689, 33, -26, 51, postcentral gyrus, precentral gyrus

4: 192, 33, 6, -4, insula

5: 148, 32, -6, -44, fusiform gyrus

6: 112, 36, -36, 39, supramarginal gyrus, superior parietal cortex

NW14:

1: 3720, 6, -57, 4, lingual gyrus, precuneus

2: 2335, -14, -48,-9, lingual gyrus, posterior cingulate

NW15:

1: 3778, 14, -9, -21, hippocampus, parahippocampal gyrus

2: 3215, -24, -4, -36, hippocampus, parahippocampal gyrus

Soll ich den migratino plot 3: 767, 16, -66, 20, n. accumbens

4: 197, 16, -66, 20, cuneal cortex, supracalcarine cortex

NW16:

1: 25138, -4, 48, 3, paracingulate gyrus, cingulate gyrus, middle frontal gyrus, frontal pole

NW17:

1: 9529, 0, 28, -14, subcallosal cortex, medial frontal cortex

NW18:

1: 8174, -3, 4, -2, putamen, amygdala

2: 7660, -3, 4, -2, putamen, amygdala

NW19:

1: 3418, 12, -96, 3, occipital pole
2: 2974, -14, -99, -14, occipital pole
3: 510, 39, -64, -48, cerebellum crus

NW20:
1: 3082, -20, 22, 2, caudate, insula
2: 2833, 51, 30, -14, orbitofrontal cortex, insula

NW21:
1: 3223, 28, -18, -32, parahippocampal gyrus, fusiform gyrus
2: 2397, -32, -10, -48, fusiform gyrus
3: 339, 48, -52, -18, inferior temporal gyrus

NW22:
1: 5026, -12, 10, -18, temporal pole, orbitofrontal cortex
2: 3925, 30, 10, -30, temporal pole, orbitofrontal cortex
3: 2263, 38, -12, -14, insula
4: 1655, -66, -20, -4, superior temporal gyrus, planum temporale
5: 631, -30, -52, -56, cerebellum VIIIa

NW23:
1: 2188, 33, -33, 40, postcentral gyrus
2: 1265, -64, -22, 26, postcentral gyrus
3: 172, -50, -27, 10, Heschl gyrus, planum temporale
4: 142, -54, -10, 2, Heschl gyrus, planum temporale
5: 141, -20, -69, -18, cerebellum VI

NW24:
1: 6802, -34, -48, -10, inferior temporal gyrus, middle temporal gyrus, fusiform gyrus
2: 4781, 57, -46, -24, inferior temporal gyrus, middle temporal gyrus
3: 406, -38, -58, -57, cerebellum VIIb, cerebellum VIIIa
4: 144, 44, 27, -9, orbitofrontal cortex
5: 32, 26, -24, orbitofrontal cortex, temporal pole

NW25:
1: 992, 21, -75, -18, cerebellum VI
2: 316, 22, -84, -38, cerebellum crus
3: 213, 56, -2, -32, middle temporal gyrus
4: 133, 33, -27, -30, fusiform gyrus
5: 126, 40, 10, 38, temporal pole

NW26:
1: 10618, -2, -20, 4, thalamus
2: 476, -21, -87, -46, cerebellum crus II
3: 462, -30, -15, 64, precentral gyrus

4: 115, -33, -96, -6, occipital pole
NW27:
1: 5041, -4, -48, 24, cingulate gyrus
2: 764, -32, -62, -60, cerebellum VIIIa
3: 188, 12, -74, 22, precuneus
NW28:
1: 4057, 51, -15, -18, middle temporal gyrus
2: 1945, -51, -16, -20, middle temporal gyrus
3: 407, 20, -80, -32, cerebellum crus
4: 292, -46, 8, -3, frontal operculum
NW29:
1: 8736, -3, 8, 58, paracingulate gyrus, juxtapositional lobule
2: 177, -27, -51, 60, superior parietal lobule
3: 125, 26, -14, 56, precentral gyrus
NW30:
1: 2731, -22, 32, 32, superior frontal gyrus, frontal pole
2: 2456, 26, 57, -8, frontal pole
3: 910, 32, -16, -10, putamen
4: 761, -30, -18, -6, putamen
5: 611, 22, -75, -30, cerebellum crus
6: 177, -16, 4, 58, superior frontal gyrus
7: 122, -38, -27, 42, postcentral gyrus

A.4 Preprocessing Brain Data

The structural images of all subjects were segmented into grey matter, white matter, and CSF using Statistical Parametric Mapping, running on MATLAB version 2018b. We used Diffeomorphic Anatomical Registration through Exponentiated Lie Algebra toolbox (DARTEL) [1] to process grey matter images. This procedure creates a sample-specific template representative of all subjects by iteratively aligning all images. Then, the template underwent non-linear registration with modulation for linear and non-linear deformations to the MNI-ICBM152 template. Subsequently, we registered each participant's grey matter map to the group template and smoothed with an 8 mm^3 isotropic Gaussian kernel.

Following the preprocessing, we computed an independent component analysis. First, all individually preprocessed grey matter maps were concatenated, creating a 4D file. An absolute grey matter threshold of 0.1 was applied to all images, ensuring that only grey matter voxels were used for the ICA. ICA was performed using the Multivariate Exploratory Linear Optimized Decomposition into Independent Components (MELODIC) method as imple-

mented in the FSL analysis package jenkinson2012fsl version 6.0. Data-driven population-based networks of grey matter covariance were derived, performing an ICA on all subjects (n=180). Therefore, this process identifies common spatial components based on the co-variation of grey matter patterns across all subjects. We allowed the process to identify 30 components (i.e., structural covariance networks, SCN), as done previously [2–4]. The results were thresholded at z = 3.5 and binarized [5, 6] to eliminate spurious results. Finally, for each participant, grey matter volume was extracted from each of the 30 morphometric networks.

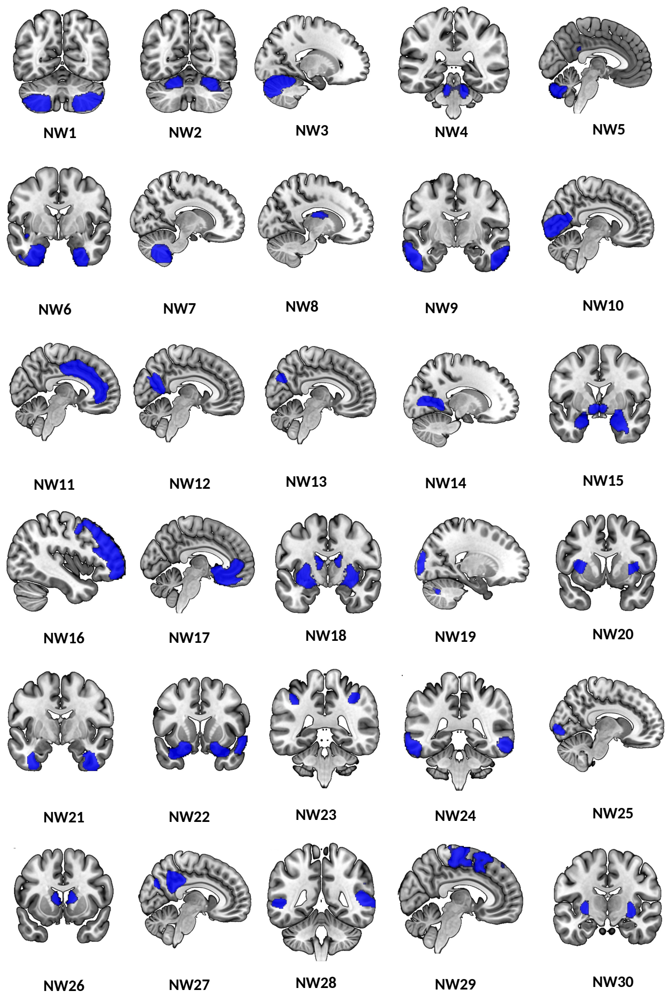

Suppl. Figure 2: **Morphometric grey matter networks.** In this figure, 30 anatomically derived morphometric networks from the ICA thresholded at Z>3.5 are displayed. Each network identifies structural grey matter covariance across all subjects.

B Results

B.1 Feature Selection

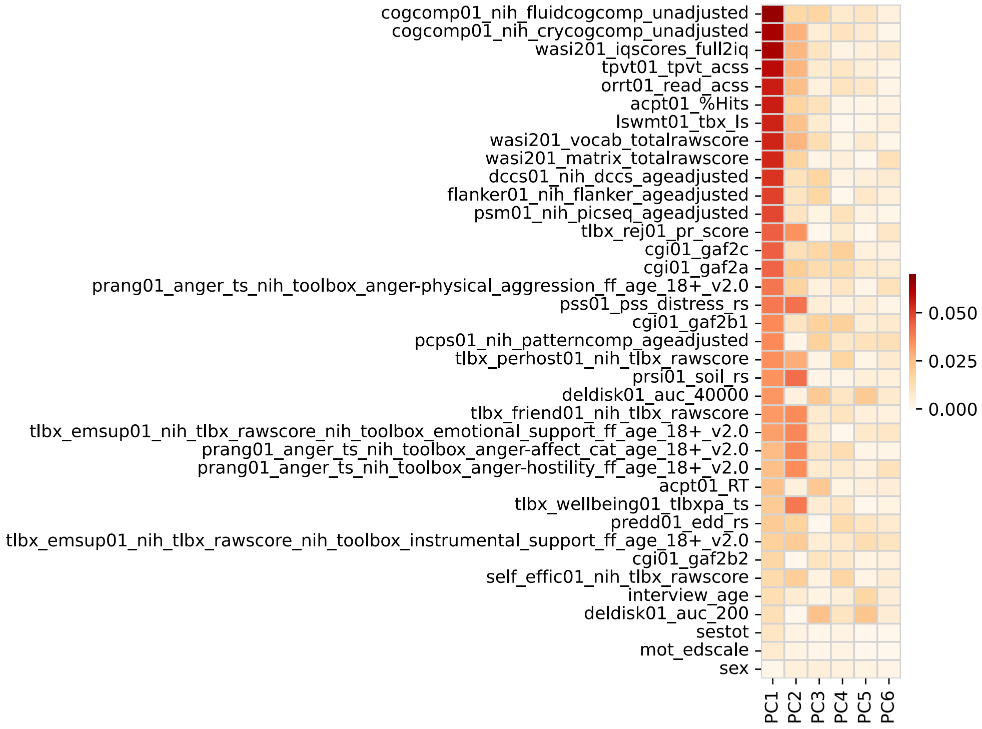

Suppl. Figure 3: **PC loadings of cognitive, perceptual and emotional data.** PCA was performed across 226 subjects on 33 features plus 4 control variables features. Displayed are PC loadings, each normalized with variance explained by the PC, making them comparable across components.

B.2 Analysis Excluding Control Subjects

Excluding controls from the clustering resulted in an overall similar distribution of subjects across the clusters (Suppl. Fig. 4), with 151/182 remaining in the same clusters. Interestingly, a part of the non-affective subjects assigned to cluster 1 were instead assigned to cluster 0 when clustering without control, possibly indicating a lower threshold for cognitive integrity. This means that individuals with more subtle cognitive deficits would also be clustered in the cognitively intact cluster. This emphasises the benefit of including controls into the clustering, as it allows true identification of those patients with intact cognition. Cluster 2 remained overall very stable (Suppl. Fig. 4c-e).

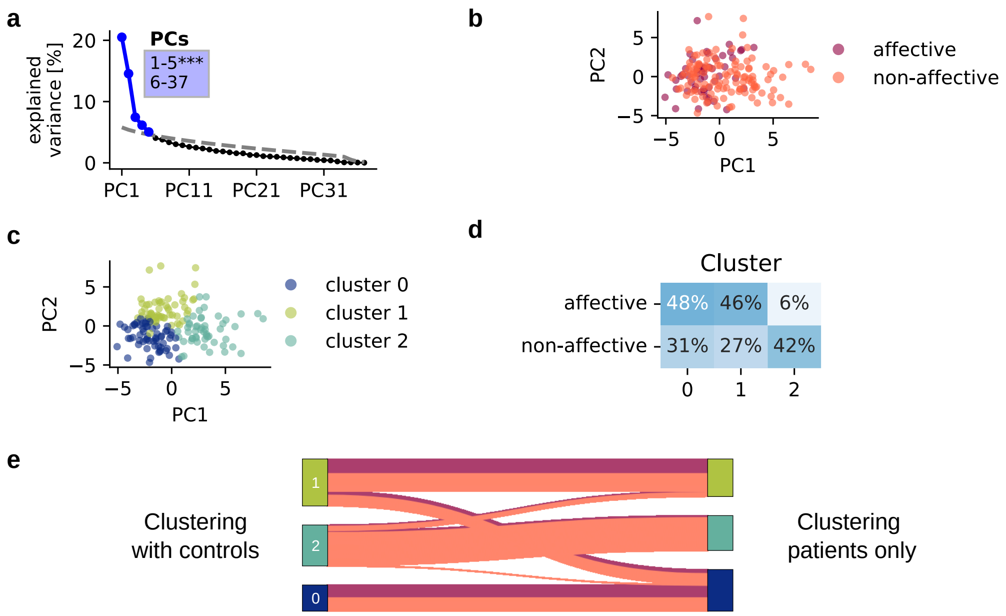

Suppl. Figure 4: **Dimensionality reduction of cognitive and brain data with patients only. (a)** Variance explained by each of the PCs in % of a PCA performed on all cognitive features and control variables across 170 patients. The first five PCs (blue) survived permutation testing ($p<0.05$, 5000 permutations). Significant components captured 53.7 % of all variance. **(b)** Cognitive data is displayed on the first two principal components and colored according to patient group affiliation. **(c)** Result of clustering that was performed on cognitive data on patients only with three clusters. Cluster affiliation of each subject is displayed on the first two PCs. Colors correspond to cluster 0, 1 or 2. **(d)** Percentage of subjects of a group in each cluster. E.g. 42 % of all non-affective subjects are in cluster 2. **(e)** Transition graph shows the transitions of individuals between clusters based on cognitive data including control (left) and cognitive of patients only (right). Subjects are divided into assigned clusters, respectively. A line connects each individual between both clusterings. The line is colour coded according to patient group (see legend in b). The graph shows the stability of the clustering.

B.3 Cluster Validation Analysis

We validated the chosen three cluster approach using a data-driven approach. Here, the numbers of clusters should be chosen to achieve low inertia and a low number of clusters. Commonly, the elbow method is employed, which identifies the point after which the improvement in the inertia value levels off. With this method, three clusters are indeed identified as the best solution (Suppl. Fig. 5a). However, other partition indices such as the partition coefficient(Suppl. Fig. 5b) and the partition entropy coefficient (Suppl. Fig. 5c), which are often chosen for fuzzy clustering [7], indicated a two cluster solution as the preferable one as compared to a three or four cluster solution. A larger partition

coefficient indicates low overlap between the clusters, hence better separation, and a larger value of partition entropy coefficient however indicates a higher overlap between cluster. As no clear statement can be made from this data-driven point of view, we felt confident to follow the approach that allowed addressing our research question most appropriately, using a predefined number of three clusters.

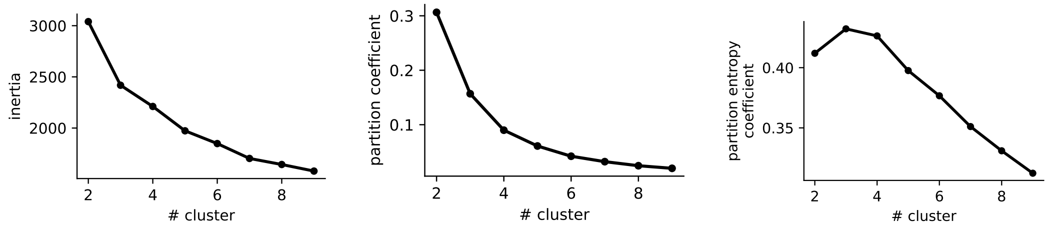

(a) (b) (c)

Suppl. Figure 5: Clustering on the five significant PCs, including controls and both patient groups, with different numbers of clusters. (a) Inertia is the sum of squared distances of samples to their closest cluster centre. The number of clusters should be chosen to achieve low inertia and a low number of clusters. (b) Partition coefficient measures the quality of a fuzzy partition. A large value means well separated clusters. (c) The partition entropy coefficient indicates fuzziness. A large value indicates high overlap between clusters.

B.4 Cluster Exploration

Suppl. Table 5: Comparison between patients in all clusters and control subjects for cognitive features. For the group comparison, a Kruskal-Wallis was used with a Dunn's test for pairwise comparisons (statistics and p-value displayed or ns - non-significant).

Feature	group comparison	cluster 0 - 1	cluster 0 - 2	cluster 0 - controls	cluster 1 - 2	cluster 1 - controls	cluster 2 - controls
cgi01_gaf2a	90.9585, <0.001	22.1896, <0.001	16.8475, <0.001	16.4083, <0.01	ns	64.5288, <0.001	57.9353, <0.001
cgi01_gaf2b1	78.1465, <0.001	14.2375, <0.001	13.3187, <0.001	17.5608, <0.001	ns	53.5162, <0.001	55.026, <0.001
cgi01_gaf2b2	ns	-	-	-	-	-	-
cgi01_gaf2c	90.8192, <0.001	27.045, <0.001	32.1442, <0.001	ns	ns	55.1229, <0.001	57.138, <0.001
cogcomp01_nih_fluidcogcomp_unadjusted	87.649, <0.001	ns	44.3611, <0.001	ns	40.0851, <0.001	15.5651, <0.001	64.5597, <0.001
cogcomp01_nih_crycogcomp_unadjusted	112.1136, <0.001	ns	63.2746, <0.001	ns	84.5395, <0.001	ns	66.9227, <0.001
dccs01_nih_dccs_ageadjusted	54.7055, <0.001	ns	27.6009, <0.001	ns	27.3806, <0.001	5.8383, <0.05	42.6152, <0.001
deldisk01_auc_200	14.7643, <0.01	ns	8.8539, <0.001	ns	8.2377, <0.01	ns	10.2426, <0.01
deldisk01_auc_40000	30.8906, <0.001	ns	17.4767, <0.001	ns	14.8141, <0.001	ns	24.9015, <0.001
flanker01_nih_flanker_ageadjusted	64.5138, <0.001	ns	29.5959, <0.001	ns	26.6117, <0.001	14.8705, <0.001	49.07, <0.001
lswmt01_tbx_ls	71.9626, <0.001	ns	32.4261, <0.001	ns	51.8415, <0.001	ns	50.0042, <0.001
orrt01_read_acss	92.0641, <0.001	ns	47.5712, <0.001	ns	68.125, <0.001	ns	59.9688, <0.001
pcps01_nih_patterncomp_ageadjusted	21.3653, <0.001	ns	6.1977, <0.05	ns	ns	10.6352, <0.01	18.9038, <0.001
prang01_anger_ts_nih_toolbox_anger-physical_aggression_ff_age_18+_v2.0	36.5683, <0.001	10.8235, <0.01	21.9381, <0.001	ns	ns	12.3361, <0.001	24.0889, <0.001
prang01_anger_ts_nih_toolbox_anger-hostility_ff_age_18+_v2.0	32.8018, <0.001	18.9004, <0.001	ns	ns	6.3975, <0.01	27.7908, <0.001	5.5897, <0.05
prang01_anger_ts_nih_toolbox_anger-affect_cat_age_18+_v2.0	37.5825, <0.001	30.3131, <0.001	3.7047, <0.05	ns	10.285, <0.001	25.3552, <0.001	ns
predd01_edd_rs	33.6906, <0.001	21.8731, <0.001	11.644, <0.01	ns	ns	20.7526, <0.001	8.3949, <0.01
prsi01_soil_rs	77.9705, <0.001	40.4617, <0.001	ns	ns	25.999, <0.001	61.7518, <0.001	11.7293, <0.001
psm01_nih_picseq_ageadjusted	61.2061, <0.001	ns	20.3563, <0.001	5.7574, <0.05	20.8384, <0.001	18.307, <0.001	51.7783, <0.001
pss01_pss_distress_rs	69.9825, <0.001	34.9998, <0.001	15.2583, <0.001	ns	8.5161, <0.05	49.2708, <0.001	25.7544, <0.001
self_effic01_nih_tlbx_rawscore	21.5996, <0.001	9.3138, <0.01	3.8954, <0.05	ns	ns	18.1286, <0.001	<0.01
tlbx_emsup01_nih_tlbx_rawscore_nih_toolbox_instrumental_support_ff_age_18+_v2.0	8.8109, <0.05	4.7407, <0.05	ns	ns	ns	7.57, <0.01	ns
tlbx_emsup01_nih_tlbx_rawscore_nih_toolbox_emotional_support_ff_age_18+_v2.0	53.608, <0.001	47.2719, <0.001	8.0771, <0.01	ns	12.9624, <0.001	30.7682, <0.001	5.0836, <0.05
tlbx_friend01_nih_tlbx_rawscore	68.0718, <0.001	38.4971, <0.001	9.7359, <0.01	ns	9.4773, <0.01	54.3892, <0.001	17.1694, <0.001
tlbx_perhost01_nih_tlbx_rawscore	32.9081, <0.001	28.8124, <0.001	6.5496, <0.01	ns	3.7249, <0.05	20.8062, <0.001	ns
tlbx_rej01_pr_score	60.2871, <0.001	44.1868, <0.001	16.6701, <0.001	ns	4.5307, <0.05	38.9551, <0.001	12.9837, <0.001
tlbx_wellbeing01_tlbxpa_ts	65.6581, <0.001	36.564, <0.001	ns	ns	25.2368, <0.001	50.8968, <0.001	6.581, <0.05
tpvt01_tpvt_acss	101.079, <0.001	ns	57.5472, <0.001	ns	77.0475, <0.001	ns	59.3365, <0.001
wasi201_vocab_totalrawscore	94.1753, <0.001	ns	51.1362, <0.001	ns	68.2115, <0.001	ns	60.9508, <0.001
wasi201_matrix_totalrawscore	69.0919, <0.001	ns	32.4274, <0.001	ns	38.7849, <0.001	6.9518, <0.05	51.9111, <0.001
wasi201_iqscores_full2iq	108.7698, <0.001	ns	57.5106, <0.001	ns	74.8416, <0.001	ns	73.4878, <0.001
acpt01_%Hits	74.1838, <0.001	ns	31.6126, <0.001	11.0381, <0.01	33.1923, <0.001	12.2936, <0.001	57.8224, <0.001
acpt01_RT	11.039, <0.05	ns	ns	ns	ns	4.5509, <0.05	10.3635, <0.001

Suppl. Table 6: Partial correlations between ten cognitive scores, three brain networks, and four clinical scores, across all patients corrected for medication; p-values are corrected for multiple comparisons.

V1	V2	estimate	statistic	p.value	adj_p.value
Fluid cognition	Cryst. cognition	0.48	5.66	0.00	0.00
Fluid cognition	Total IQ	0.50	6.03	0.00	0.00
Fluid cognition	Picture vocab. test	0.46	5.37	0.00	0.00
Fluid cognition	Oral reading recog.	0.39	4.38	0.00	0.00
Fluid cognition	Auditory attention %corr	0.59	7.53	0.00	0.00
Fluid cognition	Working memory	0.73	11.17	0.00	0.00
Fluid cognition	WASI verbal compre.	0.38	4.31	0.00	0.00
Fluid cognition	WASI matrix reason.	0.49	5.82	0.00	0.00
Fluid cognition	DCCS executive function.	0.63	8.38	0.00	0.00
Fluid cognition	PANSS total	-0.18	-1.87	0.06	1.00
Fluid cognition	PANSS positive	-0.24	-2.55	0.01	1.00
Fluid cognition	PANSS negative	-0.20	-2.07	0.04	1.00
Fluid cognition	General psychopathol.	-0.24	-2.56	0.01	1.00
Fluid cognition	NW18	-0.12	-1.23	0.22	1.00
Fluid cognition	NW29	-0.22	-2.34	0.02	1.00
Fluid cognition	NW30	-0.25	-2.72	0.01	1.00
Cryst. cognition	Total IQ	0.66	9.19	0.00	0.00
Cryst. cognition	Picture vocab. test	0.92	25.21	0.00	0.00
Cryst. cognition	Oral reading recog.	0.89	20.83	0.00	0.00
Cryst. cognition	Auditory attention %corr	0.51	6.16	0.00	0.00
Cryst. cognition	Working memory	0.54	6.72	0.00	0.00
Cryst. cognition	WASI verbal compre.	0.71	10.61	0.00	0.00
Cryst. cognition	WASI matrix reason.	0.35	3.88	0.00	0.02
Cryst. cognition	DCCS executive function.	0.36	3.96	0.00	0.02
Cryst. cognition	PANSS total	-0.29	-3.16	0.00	0.28
Cryst. cognition	PANSS positive	-0.14	-1.46	0.15	1.00
Cryst. cognition	PANSS negative	-0.41	-4.63	0.00	0.00
Cryst. cognition	General psychopathol.	-0.20	-2.09	0.04	1.00
Cryst. cognition	NW18	-0.20	-2.16	0.03	1.00
Cryst. cognition	NW29	-0.34	-3.80	0.00	0.03
Cryst. cognition	NW30	-0.42	-4.87	0.00	0.00
Total IQ	Picture vocab. test	0.64	8.65	0.00	0.00
Total IQ	Oral reading recog.	0.57	7.19	0.00	0.00
Total IQ	Auditory attention %corr	0.43	4.88	0.00	0.00
Total IQ	Working memory	0.52	6.40	0.00	0.00
Total IQ	WASI verbal compre.	0.84	16.23	0.00	0.00
Total IQ	WASI matrix reason.	0.77	12.61	0.00	0.00
Total IQ	DCCS executive function.	0.29	3.10	0.00	0.34
Total IQ	PANSS total	-0.19	-1.99	0.05	1.00
Total IQ	PANSS positive	-0.21	-2.20	0.03	1.00
Total IQ	PANSS negative	-0.40	-4.49	0.00	0.00
Total IQ	General psychopathol.	-0.14	-1.49	0.14	1.00
Total IQ	NW18	-0.12	-1.29	0.20	1.00
Total IQ	NW29	-0.39	-4.39	0.00	0.00
Total IQ	NW30	-0.39	-4.34	0.00	0.00
Picture vocab. test	Oral reading recog.	0.69	10.01	0.00	0.00

V1	V2	estimate	statistic	p.value	adj_p.value
Picture vocab. test	Auditory attention %corr	0.41	4.71	0.00	0.00
Picture vocab. test	Working memory	0.46	5.37	0.00	0.00
Picture vocab. test	WASI verbal compre.	0.70	10.06	0.00	0.00
Picture vocab. test	WASI matrix reason.	0.33	3.58	0.00	0.07
Picture vocab. test	DCCS executive function.	0.37	4.09	0.00	0.01
Picture vocab. test	PANSS total	-0.26	-2.83	0.01	0.76
Picture vocab. test	PANSS positive	-0.16	-1.63	0.11	1.00
Picture vocab. test	PANSS negative	-0.37	-4.10	0.00	0.01
Picture vocab. test	General psychopathol.	-0.15	-1.60	0.11	1.00
Picture vocab. test	NW18	-0.27	-2.88	0.00	0.65
Picture vocab. test	NW29	-0.32	-3.48	0.00	0.10
Picture vocab. test	NW30	-0.43	-4.90	0.00	0.00
Oral reading recog.	Auditory attention %corr	0.48	5.73	0.00	0.00
Oral reading recog.	Working memory	0.54	6.75	0.00	0.00
Oral reading recog.	WASI verbal compre.	0.60	7.70	0.00	0.00
Oral reading recog.	WASI matrix reason.	0.31	3.43	0.00	0.12
Oral reading recog.	DCCS executive function.	0.26	2.80	0.01	0.82
Oral reading recog.	PANSS total	-0.27	-2.93	0.00	0.56
Oral reading recog.	PANSS positive	-0.08	-0.87	0.39	1.00
Oral reading recog.	PANSS negative	-0.36	-3.98	0.00	0.02
Oral reading recog.	General psychopathol.	-0.19	-2.01	0.05	1.00
Oral reading recog.	NW18	-0.12	-1.25	0.21	1.00
Oral reading recog.	NW29	-0.32	-3.45	0.00	0.11
Oral reading recog.	NW30	-0.31	-3.41	0.00	0.13
Auditory attention %corr	Working memory	0.49	5.92	0.00	0.00
Auditory attention %corr	WASI verbal compre.	0.30	3.23	0.00	0.22
Auditory attention %corr	WASI matrix reason.	0.42	4.78	0.00	0.00
Auditory attention %corr	DCCS executive function.	0.48	5.73	0.00	0.00
Auditory attention %corr	PANSS total	-0.15	-1.53	0.13	1.00
Auditory attention %corr	PANSS positive	-0.03	-0.35	0.73	1.00
Auditory attention %corr	PANSS negative	-0.22	-2.38	0.02	1.00
Auditory attention %corr	General psychopathol.	-0.16	-1.64	0.10	1.00
Auditory attention %corr	NW18	-0.19	-2.01	0.05	1.00
Auditory attention %corr	NW29	-0.32	-3.57	0.00	0.07
Auditory attention %corr	NW30	-0.34	-3.73	0.00	0.04
Working memory	WASI verbal compre.	0.45	5.29	0.00	0.00
Working memory	WASI matrix reason.	0.42	4.78	0.00	0.00
Working memory	DCCS executive function.	0.42	4.86	0.00	0.00
Working memory	PANSS total	-0.24	-2.59	0.01	1.00
Working memory	PANSS positive	-0.19	-2.03	0.05	1.00
Working memory	PANSS negative	-0.23	-2.47	0.01	1.00
Working memory	General psychopathol.	-0.24	-2.52	0.01	1.00
Working memory	NW18	-0.14	-1.44	0.15	1.00
Working memory	NW29	-0.34	-3.79	0.00	0.03
Working memory	NW30	-0.24	-2.58	0.01	1.00
WASI verbal compre.	WASI matrix reason.	0.38	4.30	0.00	0.01
WASI verbal compre.	DCCS executive function.	0.24	2.52	0.01	1.00

V1	V2	estimate	statistic	p.value	adj_p.value
WASI verbal compre.	PANSS total	-0.24	-2.60	0.01	1.00
WASI verbal compre.	PANSS positive	-0.17	-1.83	0.07	1.00
WASI verbal compre.	PANSS negative	-0.40	-4.59	0.00	0.00
WASI verbal compre.	General psychopathol.	-0.14	-1.48	0.14	1.00
WASI verbal compre.	NW18	-0.17	-1.83	0.07	1.00
WASI verbal compre.	NW29	-0.26	-2.77	0.01	0.89
WASI verbal compre.	NW30	-0.35	-3.89	0.00	0.02
WASI matrix reason.	DCCS executive function.	0.29	3.13	0.00	0.31
WASI matrix reason.	PANSS total	-0.05	-0.48	0.63	1.00
WASI matrix reason.	PANSS positive	-0.09	-0.98	0.33	1.00
WASI matrix reason.	PANSS negative	-0.23	-2.44	0.02	1.00
WASI matrix reason.	General psychopathol.	-0.12	-1.27	0.21	1.00
WASI matrix reason.	NW18	-0.06	-0.68	0.50	1.00
WASI matrix reason.	NW29	-0.37	-4.11	0.00	0.01
WASI matrix reason.	NW30	-0.34	-3.72	0.00	0.04
DCCS executive function.	PANSS total	-0.08	-0.82	0.42	1.00
DCCS executive function.	PANSS positive	-0.14	-1.45	0.15	1.00
DCCS executive function.	PANSS negative	-0.08	-0.88	0.38	1.00
DCCS executive function.	General psychopathol.	-0.17	-1.80	0.07	1.00
DCCS executive function.	NW18	-0.08	-0.87	0.39	1.00
DCCS executive function.	NW29	-0.05	-0.54	0.59	1.00
DCCS executive function.	NW30	-0.15	-1.57	0.12	1.00
PANSS total	PANSS positive	0.38	4.24	0.00	0.01
PANSS total	PANSS negative	0.59	7.60	0.00	0.00
PANSS total	General psychopathol.	0.71	10.45	0.00	0.00
PANSS total	NW18	0.16	1.67	0.10	1.00
PANSS total	NW29	0.19	2.04	0.04	1.00
PANSS total	NW30	0.13	1.39	0.17	1.00
PANSS positive	PANSS negative	0.14	1.48	0.14	1.00
PANSS positive	General psychopathol.	0.43	4.93	0.00	0.00
PANSS positive	NW18	0.13	1.38	0.17	1.00
PANSS positive	NW29	0.20	2.09	0.04	1.00
PANSS positive	NW30	0.13	1.35	0.18	1.00
PANSS negative	General psychopathol.	0.38	4.31	0.00	0.00
PANSS negative	NW18	0.01	0.15	0.88	1.00
PANSS negative	NW29	0.02	0.24	0.81	1.00
PANSS negative	NW30	0.13	1.34	0.18	1.00
General psychopathol.	NW18	0.05	0.53	0.60	1.00
General psychopathol.	NW29	0.18	1.87	0.06	1.00
General psychopathol.	NW30	0.15	1.55	0.12	1.00
NW18	NW29	0.46	5.43	0.00	0.00
NW18	NW30	0.55	6.93	0.00	0.00
NW29	NW30	0.59	7.67	0.00	0.00
